# MolMeDB RDF: transforming a relational database about biological membranes to the RDF format to increase interoperability

**DOI:** 10.1186/s13321-026-01208-3

**Published:** 2026-06-02

**Authors:** Dominik Martinát, Jakub Juračka, Jakub Galgonek, Karel Berka

**Affiliations:** 1 Department of Physical Chemistry, Faculty of Science, Palacký University Olomouc, 17. Listopadu 12, 771 46 Olomouc, Czech Republic; 2 Department of Computer Science, Faculty of Science, Palacký University Olomouc, 17. Listopadu 12, 771 46 Olomouc, Czech Republic; 3 Institute of Organic Chemistry and Biochemistry of the Czech Academy of Sciences, Flemingovo náměstí 2, 166 00 Prague 6, Czech Republic

**Keywords:** RDF, SPARQL, Database, Membrane, Transporter, Small molecule

## Abstract

Data on molecular behaviour within biological membrane models are pivotal for predictive pharmacology, particularly for modelling bioavailability and target engagement. MolMeDB provides a curated collection of such compound-membrane interaction data. To facilitate automated reasoning and data federation, we present a semantic transformation of MolMeDB into Resource Description Framework (RDF) format, exposed through a public SPARQL endpoint. The resulting schema incorporates community-standard ontologies—including those utilised by PubChem and ChEMBL—to maximise semantic interoperability and enable complex, cross-database federated queries.

**Scientific contribution**

MolMeDB RDF provides data on interactions between molecules and membranes and molecules and transporters in machine readable graph format with links to other RDF databases. SPARQL endpoint servicing this dataset enables flexible federated querying including structural queries utilizing SACHEM.

**Supplementary Information:**

The online version contains supplementary material available at 10.1186/s13321-026-01208-3.

## Introduction

MolMeDB (Molecules on Membranes Database) is a database of interactions between small molecules and biological membranes, as well as transporter proteins [1]. This type of data is crucial for drug development because drug targets are often located within cells, and drugs must cross the cell membrane via passive transport or through the action of transporter proteins. Drugs themselves can target transporter proteins.

Molecule/membrane interaction data existed before MolMeDB, but it was scattered across various resources, including databases and scientific literature, and was recorded in a largely disorganised and inconsistent manner. MolMeDB manually and algorithmically collects experimental and computational data and provides them in a unified, standardised format. Additionally, MolMeDB presents results from in-house COSMO permeability predictions [1].

Many databases in the larger chemical and biological ecosystem describe the same molecules and other entities as MolMeDB does. For example, PubChem [2] and ChEMBL [3, 4] provide information on high-throughput assays for molecules, while UniProt [5] describes proteins, including transporters. Often, it is necessary to tap into multiple resources to gain a more complete picture, but it can be challenging to approach databases with different schemas and vocabularies.

One increasingly common approach is offering data in RDF format [6–12]. RDF, together with other technologies of the Semantic Web, such as OWL [13, 14] and SPARQL [15], allows for merging datasets and connecting corresponding entities with the same identifier, thereby using them as one large dataset. RDF data can be queried via an SPARQL endpoint, which can be provided by the database provider itself (e.g., UniProt [8]) or by a third party (e.g., IDSM [16] or a NBDC RDF portal [17]).

To enhance interoperability of molecule/membrane and molecule/transporter data, we have decided to offer MolMeDB data in RDF and make it accessible via the IDSM SPARQL endpoint.

## Construction and content

We designed an RDF schema to faithfully reflect the data in MolMeDB. The design of the MolMeDB RDF schema is built upon those of the PubChem and ChEMBL RDF schemas to ensure a high level of interoperability. This selection influenced the choice of ontologies used in the dataset (Table 1), primarily the BioAssay ontology (BAO) [18] for representing recorded interactions. To describe the types of information not stored in ChEMBL and PubChem, we have created parts of the schema de novo using terms and ontologies selected via the Ontology Lookup Service [19].

**Table 1:** Ontologies used in MolMeDB RDF

Name	Shorthand	URI prefix
RDF Schema 1.1 [20]	rdf:	http://www.w3.org/2000/01/rdf-schema#
	rdfs:	http://www.w3.org/1999/02/22-rdf-syntax-ns
XML Schema Definition Language [21]	xsd:	http://www.w3.org/2001/XMLSchema#
Simple Knowledge Organization System [22]	skos:	http://www.w3.org/2004/02/skos/core#
Semanticscience Integrated ontology [23] Chemical Information Ontology [24]	sio:	http://semanticscience.org/resource/
BioAssay Ontology [18]	bao:	http://www.bioassayontology.org/bao#
REPRODUCE-ME [25]	repr:	https://w3id.org/reproduceme#
The Information Artifact Ontology [26] Units of measurement ontology [27]	obo:	http://purl.obolibrary.org/obo/
PROV Ontology [28]	prov:	http://www.w3.org/ns/prov#
Dublin Core (DC) ontology [29]	dcterms:	http://purl.org/dc/terms/
Bibliographic Ontology [30]	bibo:	http://purl.org/ontology/bibo/
EDAM—The ontology of data analysis and management [31]	edam:	http://edamontology.org/
Experimental Factor Ontology [32]	efo:	http://www.ebi.ac.uk/efo/
MolMeDB Vocabulary	mmdbvoc:	https://rdf.molmedb.upol.cz/vocabulary#

The MolMeDB vocabulary has been created for newly defined terms using the OWL language [33]. Most terms are defined as subclasses of existing terms in other ontologies (e.g., terms for membrane measurement endpoints are subclasses of the BAO endpoint term).

### MolMeDB vocabulary

MolMeDB vocabulary contains terms needed to express MolMeDB data in RDF but not defined in biological ontologies. These terms are defined as subclasses of the BAO terms (*assay biology component*, *assay method component,* and *result*), the Unit Ontology (*unit*), and the Chemical Information Ontology (*chemical descriptor*).

**Fig. 1:**
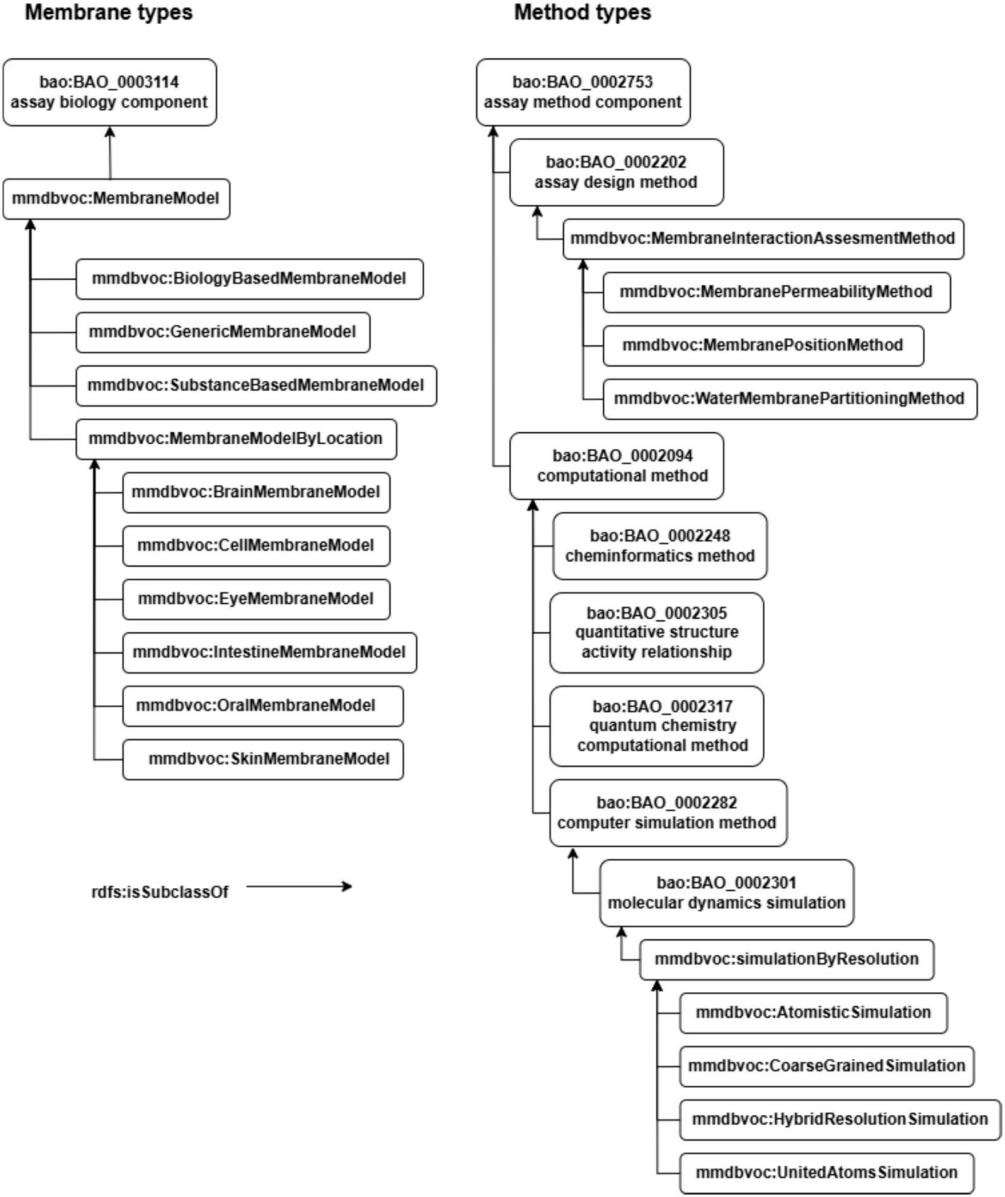
Semantic model of membranes and method types used in MolMeDB vocabulary to represent division of membranes and methods in the base MolMeDB database

Notably, the terms pertain mainly to membrane model types and methods (Fig. 1). The former are defined as a subclass of mmdbvoc:MembraneModel, a subclass of the *assay biology component* (bao:BAO_0003114). The terms themselves and their taxonomy closely follow the membrane model typology already used in MolMeDB.

We recognise membrane typology on two levels. One is whether the membrane model is based on a biological entity (*biology based substance model*) or on one or more chemical substances that mimic the behaviour of biological membranes (*substance based membrane model*). Examples of biology based models can be tissues like the eye cornea or cell culture like CACO-2 [34] or MDCK [35]. Substance based membrane models can use lipids like POPC, but can be composed of other, more generic chemicals like octanol or toluene.

The second level of membrane typology is focused on the anatomical location of the modelled membrane. We recognise cell membranes, different tissue membranes and generic membranes without any closer anatomical typology. A more sophisticated membrane model ontology is under development, but it is out of scope for this paper.

Methods are all subclasses of *assay method component* (bao:BAO_0002753). Where suitable, other BAO subclasses are utilised to create a comprehensive taxonomy of methods in MolMeDB. For the remainder, new terms have been made and added to the taxonomic tree.

Similarly to membranes, we classify methods on two levels. The first one is division into experimental, computational and simulation methods. For experimental methods, we use BAO *assay design method,* as this term's definition implies experimental methods only [18]. BAO treats simulation methods as a subclass of computational methods, but we decided to treat them as separate types to reflect classification in MolMeDB. However, we still retain this relationship in the MolMeDB vocabulary to allow inference that simulation methods are, in fact, computational.

The second level of method classification divides methods into more specific types. Experimental methods are divided by the focus of their measurements; simulation methods are divided by granularity; and computational methods are divided by their principles.

MolMeDB vocabulary also defines one data property (molmedbvoc:hasStDev) for the direct expression of the standard deviation of the assay result.

### RDF schema

We divided the entire RDF schema into 4 subdomains based on the central entity described in its unique namespace (Table 2). We have designed an RDF schema for each of these subdomains and described their correspondence to the relational dataset using R2RML language [36]. The schema was used to create a mapping for producing the MolMeDB RDF dataset via the IDSM service [16]. The RDF dataset is stored at IDSM and is queryable by its own SPARQL endpoint.

**Table 2:** MolMeDB RDF domains

Domain	Prefix name	URI prefix
Substances	mmdbmol:	https://identifiers.org/molmedb/
Substance attributes	mmdbsub:	https://rdf.molmedb.upol.cz/substance/
Membrane interactions	mmdbint:	https://rdf.molmedb.upol.cz/interaction/
Transporter interactions	mmdbtra:	https://rdf.molmedb.upol.cz/transporter/
References	mmdbref:	https://rdf.molmedb.upol.cz/reference/

The substance domain is split into substances themselves and their properties, as MolMeDB already has persistent identifiers for substances

**Fig. 2:**
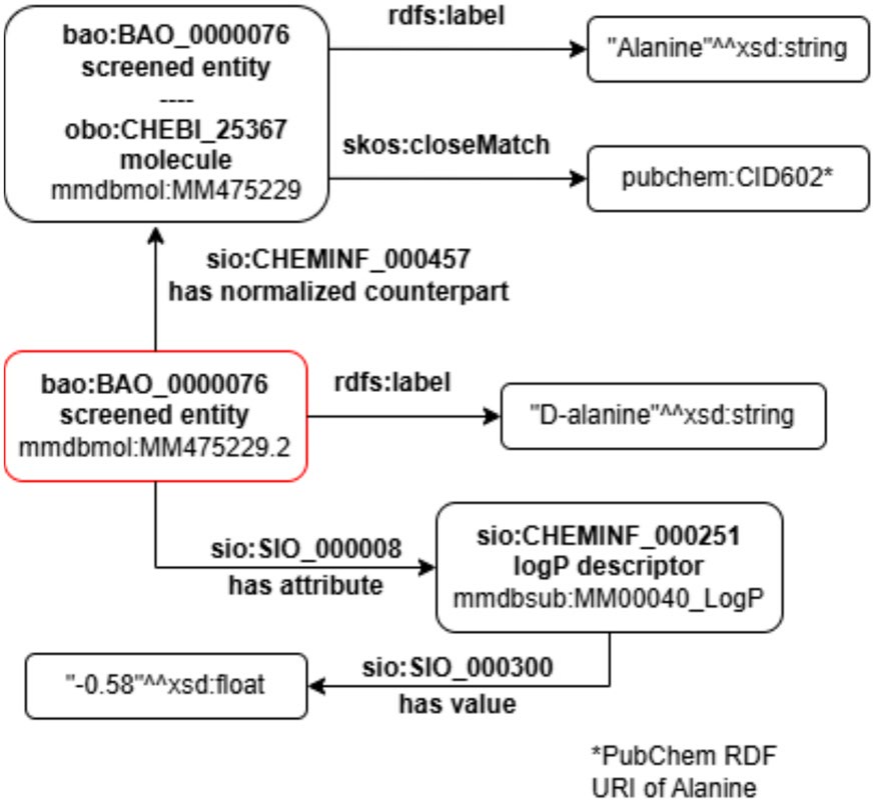
Part of the description of D-alanine (red node) and Alanine displaying the RDF schema. D-alanine has a normalized counterpart, Alanine (CID 602), which is linked to Alanine in PubChem. D-alanine also has a logP descriptor as an attribute, and logP has a value of − 0.58

The substances domain contains interacting molecules of *screened entity* (bao:BAO_0000076) type and has URIs in the form mmdbmol: <MolMeDB_identifier>. Substances are a special case in which the identifiers.org prefix is used, as the persistent identifier has already been established. They are connected to their counterparts in the RDF datasets of PubChem, ChEMBL, ChEBI, and wwPDB via skos:closeMatch, and to their properties via *has attribute* (sio:SIO_000008) predicates (Fig. 2). Substance attributes are distinct nodes with assigned type and URI, which take the form mmdbsub:<id>_<property name> (e.g., mmdbsub:MM00040_Molecular_Weight for molecular weight of caffeine). Attribute nodes are connected to their values by *has value* (sio:SIO_000300) predicate and by *has unit* (sio:SIO_000221) to the unit used, where applicable.

Some substances can exist in multiple forms that differ in charge, orientation, or other physical properties. These forms are treated as separate molecules. To identify molecular forms with their basic form, we link them using *has normalised counterpart* (sio:CHEMINF_000457) predicate. Substances already in their basic form link to themselves and have an additional *molecule* (obo:CHEBI_25367), which is explicitly defined as an electric neutral entity [37].

Obsolete molecule identifiers are not removed from the dataset. Instead, they are connected to the replacing record by *term replaced by* (obo:IAO_0100001) predicate and have assigned obsolescence reason as terms merged (<obsolete_molecule> obo:IAO_0000231 obo:IAO_0000227).

The membrane interactions subdomain describes membrane interactions themselves, including experimental conditions, methods, and the membranes involved.

**Fig. 3:**
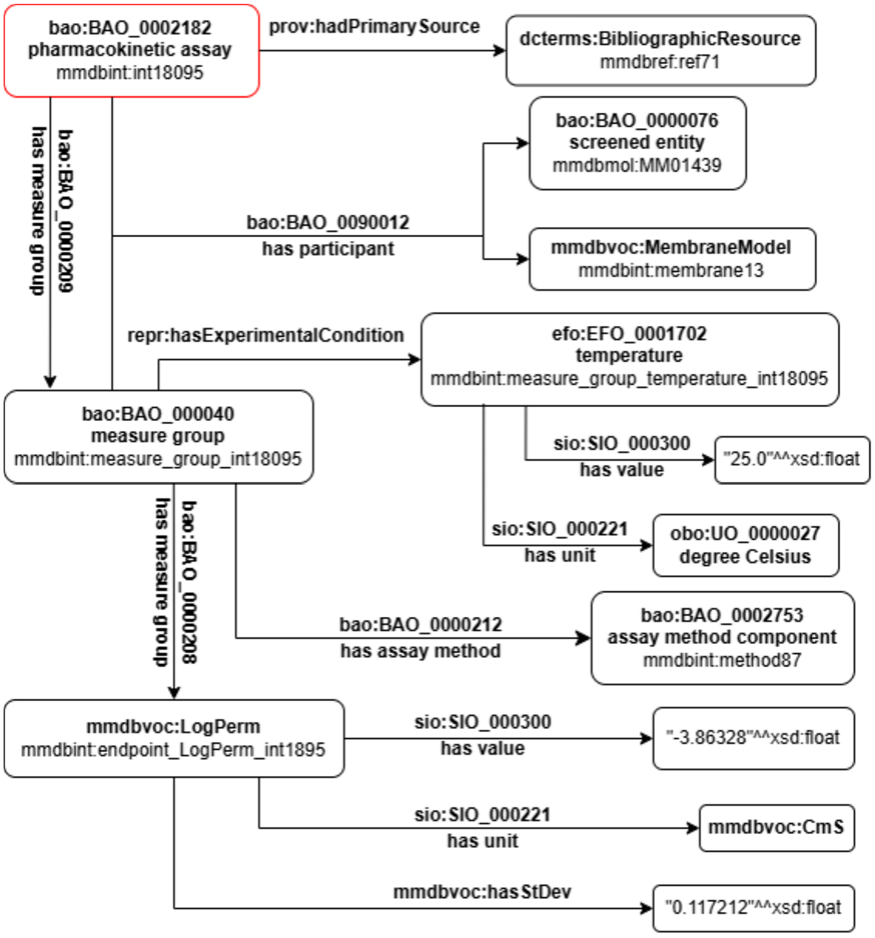
Diagram of a subgraph describing molecule–membrane interaction assay (red node). Assay is connected to its primary source and measure group. Both the assay and measure group are connected to the participants: the SL-PC membrane and the benzene molecule. The measure group is connected to the assay method and experimental condition (temperature). The measure group is connected to the measurement result in the shape of an endpoint (LogP), which has its own value, unit and standard deviation

The description of membrane interaction assays follows the BioAssays ontology, in which each bioassay node is connected to one or more measure group nodes, and each measure group node is connected to one or more endpoint nodes (Fig. 3). The data structure in MolMeDB ensures that each bioassay is connected to precisely one measure group node.

URIs of bioassays have the structure mmdbint:int<id>, their corresponding measure group mmdbint:measure_group_int_<id>, and corresponding endpoint mmdbint:endpoint_<type>_int<id>. Bioassays using fluorescent properties are treated separately, but they follow the same schema with the same predicates and datatypes; their URIs use 'fluo' instead of 'int' in their suffixes.

Membrane assays have type *pharmacokinetic assay* (bao:BAO_0002182) and are connected to participating membranes and molecules via *has participant* (bao:BAO_0090012) predicate. We use two types of links to record data provenance. For primary reference, we use prov:hadPrimarySource, and prov:wasQuotedFrom to identify the source from which the data for MolMeDB were obtained. For example, it is common for data published in a scientific paper to be mined from a database; in this case, the paper is considered a primary reference, and the database is regarded as a source. However, data obtained from a paper directly would have the same primary reference as the source. 

*Measure groups* (type bao:BAO_0000040) are connected to relevant membranes and molecules in the same way as membrane assay nodes. They are also connected to the assay method via *has assay method* (bao:BAO_0000212) and to experimental conditions via repr:hasExperimentalCondition predicates. They can also be linked to comments using rdfs:comment predicate.

Endpoints can be classified by the value measured (Table 3). Their values are expressed by the predicate *has value* (sio:SIO_000300), and each type has an assigned unit (predicate sio:SIO_000221). In cases where MolMeDB contains information on the measurement standard deviation, the endpoint is connected to its value via the mmdbvoc:hasStDev predicate.

**Table 3:** Endpoint types for molecular–membrane interactions

Endpoint type URI	Definition
mmdbvoc:DepthOfMinima	Depth of potential energy minima within the membrane
mmdbvoc:LogK	Logarithm of partition coefficient membrane/water
mmdbvoc:LogPerm	Logarithm of membrane permeability
mmdbvoc:PenetrationBarrier	Energy barrier for membrane penetration
mmdbvoc:PositionOfMinima	Position of potential energy minima along the membrane normal from the membrane center
Fluorescent properties	
mmdbvoc:AbsorptionWavelength	Peak absorption wavelength
mmdbvoc:ContactAngle	Contact angle
mmdbvoc:FluorescenceLifetime	Fluorescence lifetime
mmdbvoc:FluorescenceWavelength	Peak fluorescence wavelength
mmdbvoc:QuantumYield	Quantum yield

Experimental conditions (Fig. 3) are either the *charge* of the molecule (type sio:CHEMINF_000120), *temperature* (type efo:EFO_0001702), or *pH* (type sio:SIO_001089) of the measured system. Both are connected to their values (predicate sio:SIO_000300). Temperature is expressed in degrees Celsius (<temp>sio:SIO_000221 obo:UO_0000027).

*Membrane models* (mmdbvoc:MembraneModel) and *assay methods* (bao:BAO_0002753) are described in a simplified manner. Each is also more narrowly categorized by its respective subtype from the MolMeDB vocabulary. Their URIs take the form mmdbint:membrane<id> and mmdbint:method<id>, respectively. Both have names and abbreviations expressed via rdfs:label predicate. Membranes are, in some cases, also manually connected to their molecular components in ChEBI using *has part* (bao:BAO_0090004) predicate.

The transporter assays subdomain focuses on *transporter assays* (type bao:BAO_0003008) and proteins and is structured very similarly to the membrane assays domain. URIs of transporter assays take the form mmdbtra:tra<id>, and their measure group and endpoint URIs are formed in the same manner as in the membrane assay subdomain. Other differences from membrane assays include omitting methods and experimental conditions, and using a transporter protein rather than a membrane.

Transporter assay endpoints can be divided into two categories: 1) concentration-dependent endpoints and 2) categorical endpoints. Concentration-dependent endpoints describe effects of molecule concentrations (*pIC50* bao:BAO_0000199, *pEC50* bao:BAO_0002583, *pKi* bao:BAO_0190004, *pKm* mmdbvoc:PKm) on the transporter in question.

**Fig. 4:**
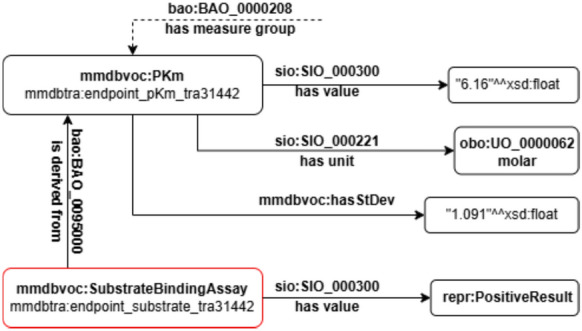
Diagram of a subgraph showing a derived endpoint (red node) in the MolMeDB RDF schema. In this case, the derived endpoint represents that we interpret the pKm endpoint value as telling that the substance is a substrate of the transporter protein, which was the target of the original assay

Categorical endpoints (Fig. 4) are derived from concentration-related ones, and they represent our interpretation of the result in terms of whether the substance acts as an inhibitor or a substrate. For inhibitors, we use the mmdbvoc:InhibitionAssay type, and consider a substance an inhibitor if its pKi or pIC50 value is greater than 0.5. For the substrate, we use the mmdbvoc:SubstrateBindingAssay type, and consider a substance a substrate if its pKm or pEC50 is greater than 0.5. The fact that a substance is interpreted as an inhibitor or a substrate is denoted by repr:PositiveResult as endpoint value. The opposite case uses repr:NegativeResult instead.

**Fig. 5:**
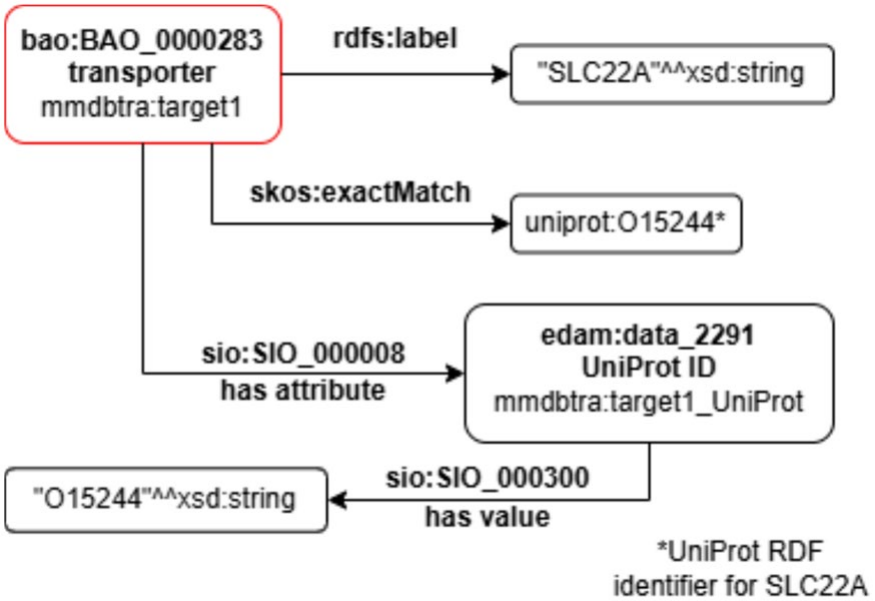
Graph of the RDF description of the SLC22A1 transporter (red node). Transporter is connected to its name and its UniProt RDF counterpart, and has a UniProt ID attribute whose value is the UniProt identifier

Transporter proteins (Fig. 5) use URIs of mmdbtra:target<id> pattern and are of *transporter* (bao:BAO_0000283) type. They link to their gene name (rdfs:label predicate) and UniProt counterpart (skos:exactMatch predicate). The transporter node is connected to its *UniProt ID* (edam:data_2291 type) using the *has attribute* (sio:SIO_000008) predicate. A more detailed description of transporter proteins is available in the UniProt database, accessible via federated querying.

The References subdomain describes assay sources. The source can be either a publication, a database, or an in-house calculation, which is itself treated as having a source in the MolMeDB database.

**Fig. 6:**
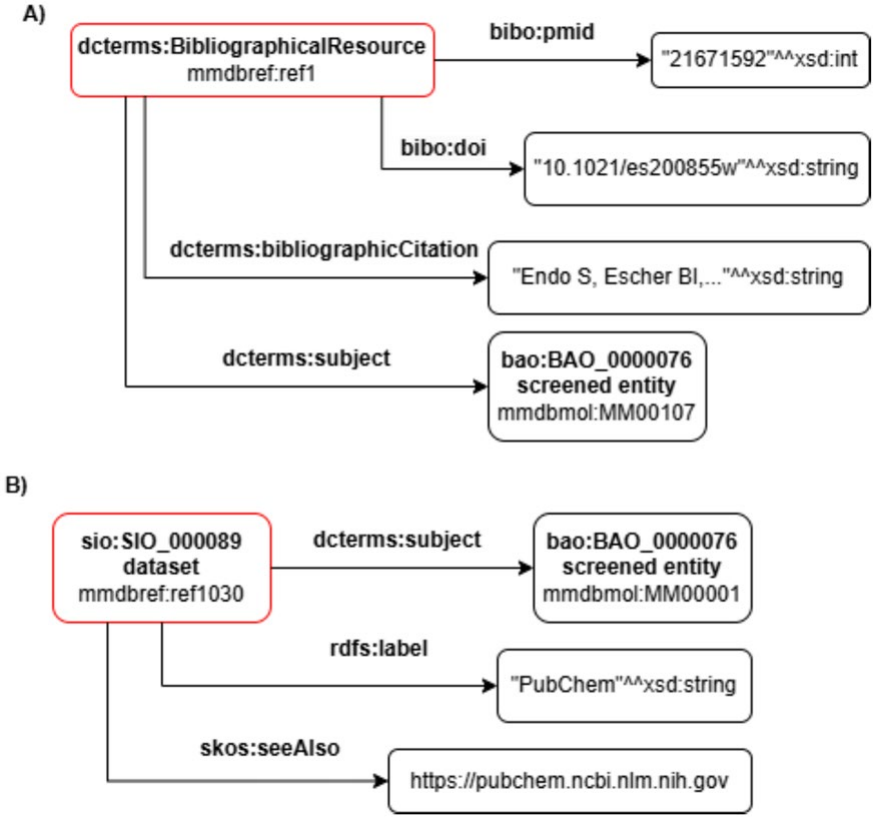
Subgraphs of references subdomain. (**A** red node) Publication is described by DOI and PMID identifiers. It is also described using a classical bibliographic citation when available. It is also connected to the described entities (molecules, membranes and transporters). (**B** red node) Dataset is similarly connected to described entities but instead of identifiers, it uses name of dataset and URL where it is available

Publications (typed dcterms:BibliographicResource) are linked to a digital object identifier and/or PubMed ID identifier by bibo:doi and/or bibo:pmid predicate, respectively. Both are string literals. There is also a classical citation provided as a string literal linked by dcterms:bibliographicCitation predicate (Fig. 6).

Databases (sio:SIO_000089 *dataset* type) are linked to their URLs using the rdfs:seeAlso predicate; assays from in-house calculations use the MolMeDB database as their source, and their names are expressed using the rdfs:label predicate. Both publications and databases are linked to relevant substances, membranes, and transporter proteins by dcterms:subject predicate.

## Utility and discussion

MolMeDB RDF provides a complement to more general databases such as PubChem or ChEMBL within the life science semantic data system. It provides specialised data on molecule-membrane and molecule transporter interactions with ontology terms and an RDF schema designed for its particular needs.

MolMeDB RDF dataset is available in RDF/XML, Turtle, and N-Triples serializations. The latest version is available for free download [38]. MolMeDB RDF is regularly updated to stay consistent with the original MolMeDB.

MolMeDB RDF can be queried using a SPARQL endpoint [39]. This endpoint is provided by IDSM, which also enables the use of its SACHEM functionality for structural queries.

A detailed description of access to MolMeDB RDF and other use options is available in the RDF section of the MolMeDB documentation [40], and summary statistics of the dataset are available in Additional file 1 Table S1.

MolMeDB vocabulary terms already use hash dereference to the vocabulary document in RDF/XML format. URI dereference of other MolMeDB terms is planned together with MolMeDB web application update. We plan to provide access to RDF documents in Turtle, N-triples and RDF/XML formats describing the entity URI pertains to.

### Federated querying

**Fig. 7:**
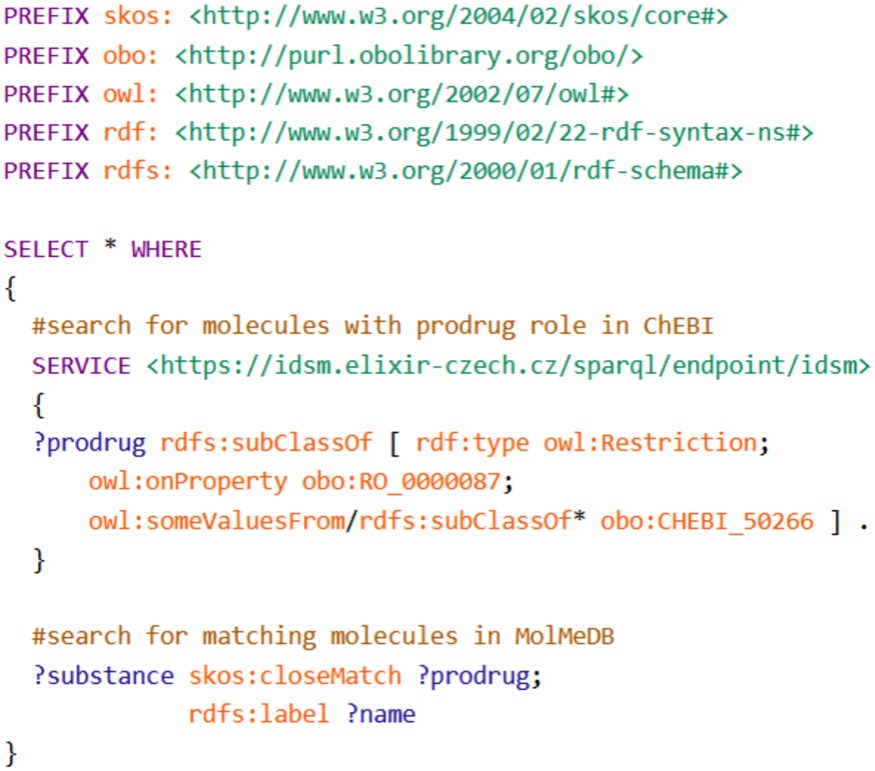
Federated SPARQL query for molecules in MolMeDB RDF that are listed as a prodrug in ChEBI RDF

External identifiers from MolMeDB are used to construct URIs of corresponding entities from other RDF datasets. Connections to small molecules and transporters in the other dataset are explicitly expressed via the exact match property. This enables the relatively easy querying of multiple RDF datasets simultaneously, as illustrated in the example below (Fig. 7). Next to the flexibility provided by SPARQL language, we see this as the main contribution of MolMeDB RDF. Both capabilities can be used to answer complex questions. For example we have used this to obtain permeabilities of drugs that act as human ABC transporter inhibitors for different membrane models [see Additional file 1 Query S2].

**Fig. 8:**
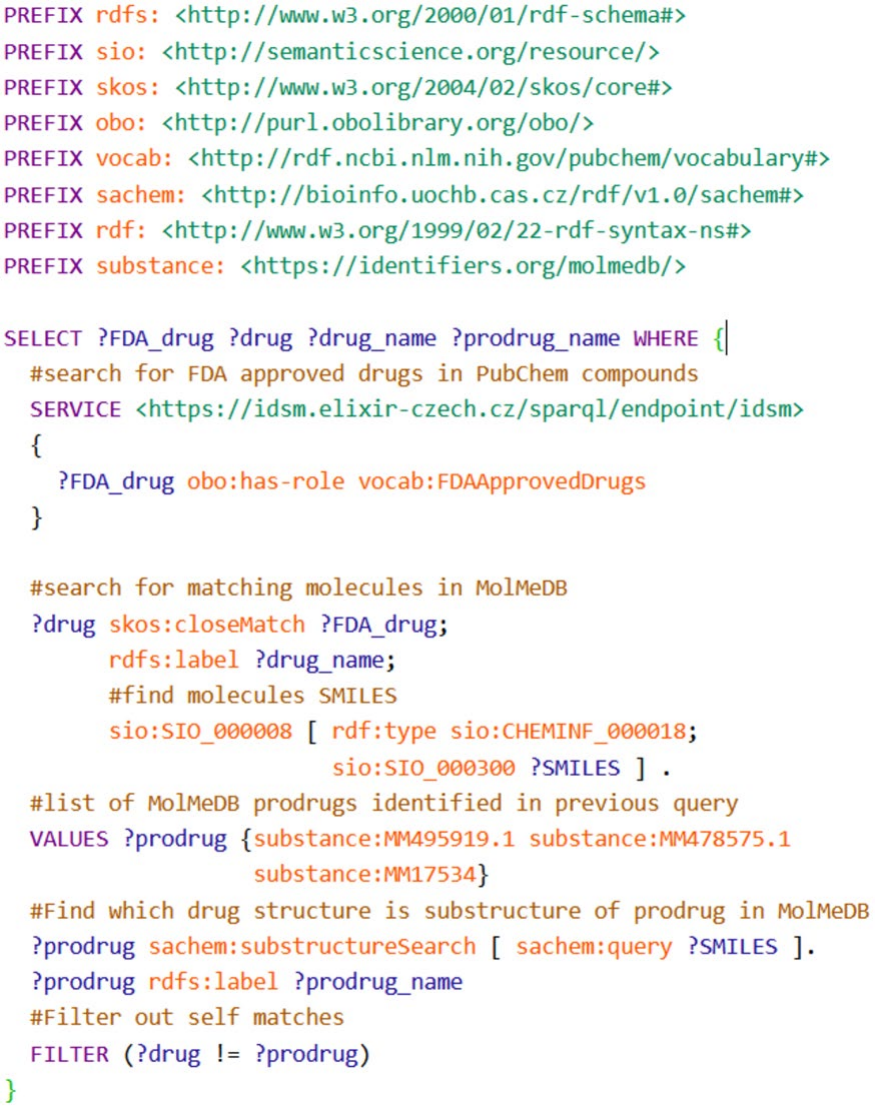
Federated SPARQL query for FDA-approved drugs whose structure can be found as a substructure in a subset of selected molecules from the previous query result. Substructure search is done via SACHEM functionality

This can be used in conjunction with SACHEM to create queries that leverage structured information (Fig. 8).

## Conclusion

By providing its dataset in RDF, MolMeDB adds data on molecule-membrane interactions to the wider chemical semantic web created by the ecosystem of chemical RDF databases, such as PubChem RDF. This helps to set our chemical knowledge into a more complete context.

Accessibility of MolMeDB RDF by SPARQL endpoint has two main advantages: 1) wide freedom for chemical software developers to customize their queries to the database, and 2) possibility of using a larger part of the semantic web via federated querying.

The aim of achieving greater interoperability with other RDF databases significantly influenced our choice of ontologies; however, we found a distinct lack of ontological descriptions of biological membranes, which led to the aforementioned definition of our own terms. This, however, is a crude solution in some cases, and future versions of MolMeDB RDF will likely use different terms or ontologies, depending on developments in the field of the semantic description of membranes.

## Supplementary Information

Supplementary material 1.

## Data Availability

MolMeDB RDF dataset is accessible in turtle, N-triples and RDF/XML formats at 10.5281/zenodo.18632779. SPARQL endpoint servicing MolMeDB RDF dataset is at https://idsm.elixir-czech.cz/sparql/endpoint/molmedb. Original MolMeDB database and documentation can be found at https://molmedb.upol.cz. Additional file 1 provides another example of MolMeDB RDF use in the shape of SPARQL queries with explanation and MolMeDB RDF summary statistics.
