# Supplementary material for: MolMeDB RDF: transforming a relational database about biological membranes to the RDF format to increase interoperability
Source: J Cheminform. 2026 Jun 2;18:111. doi: 10.1186/s13321-026-01208-3 (PMC13449323; doi:10.1186/s13321-026-01208-3)
Supplement: Supplementary file 1 [file 13321_2026_1208_MOESM1_ESM.docx]

**Additional file 1 for
MolMeDB RDF: transforming a relational database about biological membranes to the RDF format to increase interoperability**

**Dominik Martinát^1^, Jakub Juračka^1,2^, Jakub Galgonek^3^, Karel Berka^1,3^**

1 Department of Physical Chemistry, Faculty of Science, Palacký University Olomouc, 17. listopadu 12, 771 46 Olomouc, Czech Republic

2 Department of Computer Science, Faculty of Science, Palacký University Olomouc, 17. listopadu 12, 771 46 Olomouc, Czech Republic

3 Institute of Organic Chemistry and Biochemistry of the Czech Academy of Sciences, Flemingovo náměstí 2, 166 00 Prague 6, Czech Republic

**Table S1.** Summary statistics of the MolMeDB dataset at the date of submission (April 2026). These are expected to grow as the MolMeDB database and subsequent updates to the MolMeDB RDF are added.

| **basic statistics** | |
| --- | --- |
| triplets | 34019186 |
| types | 61 |
| predicates | 28 |
| **class statistics** | |
| molecular entities | 476970 |
| membrane assay | 974454 |
| methods | 57 |
| membranes | 49 |
| transporter assays | 4472 |
| transporters | 208 |
| primary sources | 3220 |
| **outlinks** | |
| molecule to chebi | 2132 |
| molecule to chembl | 20703 |
| molecule to pdb | 857 |
| molecule to pubchem | 78359 |
| membrane components to chebi | 30 |
| transporters to UniProt | 208 |
| total outlinks | 102289 |

**Query S2.** We have used the capabilities of the MolMeDB RDF SPARQL endpoint to search for drug permeabilities in different membrane models for drugs that act as human ABC transporter inhibitors. Human ABC transporters have been identified using UniProt RDF and matching transporters, and their inhibitors have been found in MolMeDB RDF. Which of these inhibitors are drugs has been decided using ChEBI RDF. Their permeabilities in different membrane models have been found in MolMeDB.

SPARQL endpoint: <https://idsm.elixir-czech.cz/sparql/endpoint/molmedb>

```
PREFIX bao: <http://www.bioassayontology.org/bao#>
PREFIX ebi: <http://rdf.ebi.ac.uk/dataset/>
PREFIX GO: <http://purl.obolibrary.org/obo/GO_>
PREFIX mmdbvoc: <https://rdf.molmedb.upol.cz/vocabulary#>
PREFIX obo: <http://purl.obolibrary.org/obo/>
PREFIX owl: <http://www.w3.org/2002/07/owl#>
PREFIX rdf: <http://www.w3.org/1999/02/22-rdf-syntax-ns#>
PREFIX rdfs: <http://www.w3.org/2000/01/rdf-schema#>
PREFIX repr: <https://w3id.org/reproduceme#>
PREFIX sio: <http://semanticscience.org/resource/>
PREFIX skos: <http://www.w3.org/2004/02/skos/core#>
PREFIX up: <http://purl.uniprot.org/core/>

SELECT DISTINCT ?prot ?ChEBI_mol ?membrane ?LogPerm WHERE {
	# Human ABC transporters
	SERVICE <https://sparql.uniprot.org/sparql> {
		?prot up:classifiedWith GO:0140359 ;
       		  up:organism/up:scientificName "Homo sapiens" }

	# inhibitors of human ABC transporters with ChEBI match
	?assay bao:BAO_0090012 [ a bao:BAO_0000283 ;
    						skos:exactMatch ?prot ] ;
           bao:BAO_0090012 ?mol ;
           bao:BAO_0000208/^bao:BAO_0095000 [a mmdbvoc:InhibitionAssay ;
                           sio:SIO_000300 repr:PositiveResult ] .
	?mol  a bao:BAO_0000076 ;
          	rdfs:label ?name ;
           skos:closeMatch ?ChEBI_mol .

	# ChEBI molecules classified as drugs
	SERVICE <https://idsm.elixir-czech.cz/sparql/endpoint/idsm> { GRAPH ebi:chebi {
        	?ChEBI_mol rdfs:subClassOf
          		[ owl:someValuesFrom/rdfs:subClassOf* obo:CHEBI_23888 ;
            	  rdf:type owl:Restriction ;
            	  owl:onProperty obo:RO_0000087 ] ; }}

	?perm_assay bao:BAO_0090012 ?mol ;
  			    bao:BAO_0090012 [a mmdbvoc:MembraneModel ;
       						     rdfs:label ?membrane ];
     		    bao:BAO_0000208 [ a mmdbvoc:LogPerm ;
     							  sio:SIO_000300 ?LogPerm ] .
}
```

**Query S3**: an example of how to query SPARQL endpoint for computational/experimental data

```
PREFIX mmdbvoc: <https://rdf.molmedb.upol.cz/vocabulary#>
PREFIX bao: <http://www.bioassayontology.org/bao#>
PREFIX sachem: <http://bioinfo.uochb.cas.cz/rdf/v1.0/sachem#>

SELECT DISTINCT * WHERE {
  ?assay bao:BAO_0000208 ?endpoint ;
         bao:BAO_0000212 ?method .

  #method type
  #bao:BAO_0002202 - assay design method (experimental)
  #bao:BAO_0002094 - computational method
  #bao:BAO_0002301 - simulation method
  ?method a bao:BAO_0002202 .

  #endpoint type
  #mmdbvoc:LogPerm
  #mmdbvoc:LogK
  ?endpoint a mmdbvoc:LogK
}
```
